# Protein expression of prognostic genes in primary melanoma and benign nevi

**DOI:** 10.1007/s00432-021-03779-0

**Published:** 2021-11-10

**Authors:** T. Gambichler, J. Elfering, T. Meyer, S. Bruckmüller, E. Stockfleth, M. Skrygan, H. U. Käfferlein, T. Brüning, K. Lang, D. Wagener, S. Schröder, M. Nick, L. Susok

**Affiliations:** 1 Skin Cancer Center, Department of Dermatology, Ruhr-University Bochum, Bochum, Germany; 2 Institute for Prevention and Occupational Medicine of the German Social Accident Insurances, Ruhr-University Bochum (IPA), Bochum, Germany; 3 Pathology/Labor Lademannbogen MVZ GmbH, Hamburg, Germany

**Keywords:** Cutaneous melanoma, Gene expression, Protein expression, Prognostic assay, Biomarker, MelaGenix

## Abstract

**Purpose:**

To evaluate the protein expression characteristics of genes employed in a recently introduced prognostic gene expression assay for patients with cutaneous melanoma (CM).

**Methods:**

We studied 37 patients with CM and 10 with benign (melanocytic) nevi (BN). Immunohistochemistry of primary tumor tissue was performed for eight proteins: COL6A6, DCD, GBP4, KLHL41, KRT9, PIP, SCGB1D2, SCGB2A2.

**Results:**

The protein expression of most markers investigated was relatively low (e.g., DCD, KRT9, SCGB1D2) and predominantly cytoplasmatic in melanocytes and keratinocytes. COL6A6, GBP4, and KLHL41 expression was significantly enhanced in CM when compared to BN. DCD protein expression was significantly correlated with COL6A6, GBP4, and KLHL41. GBP4 was positively correlated with KLHL41 and inversely correlated with SCGB2B2. The latter was also inversely correlated with serum S100B levels at time of initial diagnosis. The presence of SCGB1D2 expression was significantly associated with ulceration of the primary tumor. KRT9 protein expression was significantly more likely found in acral lentiginous melanoma. The presence of DCD expression was less likely associated with superficial spreading melanoma subtype but significantly associated with non-progressive disease. The absence of SCGB2A2 expression was significantly more often observed in patients who did not progress to stage III or IV.

**Conclusions:**

The expression levels observed were relatively low but differed in part with those found in BN. Even though we detected some significant correlations between the protein expression levels and clinical parameters (e.g., CM subtype, course of disease), there was no major concordance with the protective or risk-associated functions of the corresponding genes included in a recently introduced prognostic gene expression assay.

## Introduction

Cutaneous melanoma (CM) is one of the most aggressive types of skin malignancies, accounting for about 75% of skin-cancer-related mortality (Schadendorf and Hauschild 2014). A characteristic feature of CM is the ability to metastasize at early stages of tumor progression. Within the past years, however, rapidly evolving immunotherapy and targeted therapy modalities have significantly extended the life expectancy of patients with advanced CM (Zhu et al. 2016; Eggermont and Robert 2011). These novel treatment regimens have recently found entry into the adjuvant therapeutic setting (Weber et al. 2017; Long et al. 2017). The latter treatments currently represent new therapeutic approaches for patients with positive sentinel lymph node biopsy and even stage II patients are currently under investigation in this context (Weber et al. 2017; Long et al. 2017).

Broad application in adjuvant treatment setting of clinically tumor-free patients is hampered by side effects and associated with high costs of the aforementioned new therapies (Weber et al. 2017; Long et al. 2017). Therefore, future treatment strategies have to be precise and early in recognition of patients at high risk of melanoma recurrence. Thus, prognostic biomarkers complementing conventional staging systems are required to enable a more accurate identification of “true” high-risk patients who actually need adjuvant treatment. We and others have previously investigated and validated a gene expression profile score in primary CM and adjacent stroma, consisting of eight genes (in addition to three housekeeping genes) predicting patient survival independently of the American Joint Committee on Cancer (AJCC) stage (Gambichler et al. 2021a; Amaral et al. 2020; Brunner et al. 2013, 2018; Gershenwald et al. 2017). In fact, the MelaGenix® assay appears to provide significant prognostic information. In stages II and III a larger group of patients with a low-risk score could be detected, and for these patients, adjuvant treatment could be considered unnecessary. Hence, the use of the MelaGenix® test appears to be suitable for the selection of patients with adjuvant treatment regimens, preventing side effects in low-risk patients and thus reducing costs in this context (Gambichler et al. 2021a). However, the protein expression profiles of the MelaGenix® assay genes have not yet been characterized in melanocytic skin lesions.

In the present study, we aimed to evaluate for the first time the protein expression profiles of the genes employed in the prognostic MelaGenix® gene expression assay in primary CM and compare it with expression profiles found in benign (melanocytic) nevi (BN).

## Material and methods

### Study population

We searched through our database for CM and BN of patients treated in the year 2018 in the Skin Cancer Center, Ruhr-University Bochum. All tumors were diagnosed by two experienced dermato-histopathologists according to standard histopathological criteria for CM (Schadendorf and Hauschild 2014). We only included patients of whom the formalin-fixed paraffin-embedded (FFPE) tissue of the primary tumor was available. Complete clinical work-up, staging, follow-up and treatment were performed corresponding to current CM’s guidelines (Schadendorf and Hauschild 2014; Gershenwald et al. 2017). Clinical data were collected by chart review. As controls, we also recruited patients with BN. For further analysis, we evenly stratified in low-risk (≤ 2 mm tumor thickness) and high-risk (> 2 mm tumor thickness) melanomas and included cases with available tumor tissue and sufficient clinical data details only.

### Immunohistochemistry and microscopic evaluation

Immunohistochemistry was performed in accordance with the manufacturer’s recommendations. Briefly, sections of formalin-fixed, paraffin-embedded (FFPE) tissue were dried overnight at 37 °C, deparaffinized in Rotihistol (Carl Roth, Karlsruhe, Germany) and subsequently hydrated through a graded alcohol series. For immunostaining, we used primary antibodies as follows: COL6A6 (collagen type VI alpha 6 chain) [Abcam, Waltham, USA, Cat# ab150926, dilution 1:100]; DCD (dermcidin) [Sigma, Taufkirchen, Germany, Cat# HPA063967, dilution 1:2000]; GBP4 (guanylate binding protein 4) [Abcam, Waltham, USA, Cat# ab232693, dilution 1:750]; KLHL41 (kelch-like family member 41) [Sigma, Taufkirchen, Germany, Cat# HPA021753, dilution 1:500]; KRT9 (keratin 9) [Biozol, Eching, Germany, Cat# DF9001, dilution 1:200]; PIP (prolactin-induced protein) [Sigma, Taufkirchen, Germany, Cat# HPA009177, dilution 1:200]; SCGB1D2 (lipophilin B) [NOVUS, Centennial, USA, Cat# NBP1-81304, dilution 1:500]; SCGB2A2 (mammaglobin A) [Cell Marque, Rocklin, USA, Cat# 280C-14, dilution1:100].

Visualization was performed using the Dako REAL™ Detection System, Alkaline Phosphatase/RED, Rabbit/Mouse (K5005, Dako Agilent; Santa Clara, CA) according to the manufacturer`s protocol. For nuclear counterstaining, specimens were incubated in hematoxylin (S202084, Dako Agilent) for 1 min followed by a 5-min-incubation in tap water. Finally, samples were processed through a series of ascending alcohol concentrations and mounted with Entellan (Merck, Darmstadt, Germany). For microscopic analysis, stained slides were scanned at 40× magnification using the Nanozoomer Whole Slide Scanner from Hamamatsu (Hamamatsu, Herrsching am Ammersee, Germany). The images were evaluated using the viewer software NDP.view2 (Hamamatsu Photonics, Germany). As previously reported, H-score quantification was performed by multiplying the percentage of positive cells (0–100%) by the staining intensity (0 = none; 1 = slight; 2 = moderate; 3 = strong) and totalization of data (total range 0–300) (Gambichler et al. 2021b). We also categorized immunostaining in a negative (immunoreactivity = 0) and a positive (immunoreactivity > 0) group.

### Statistics

Data analysis was performed using the statistical package MedCalc Software version 20.008 (MedCalc, Ostend, Belgium). The distribution of data was assessed by the D’Agostino-Pearson test. For non-normally distributed data, the median and range were calculated. Data were analyzed where appropriate using the Chi^2^ test, Spearman correlation procedure, and Mann–Whitney test. *P* values of < 0.05 were considered significant.

## Results

Study population consisted of 37 patients with CM, including 18/37 (48.6%) women and 19/37 (51.4%) men at the median age of 71 years (28–91 years). The primary CM investigated consisted of 18/37 (48.6%) superficial spreading melanomas (SSM), 5/37 (13.5%) nodular melanomas (NM), 6/37 (16.2%) lentigo maligna melanomas, and 8/37 (21.6%) acral lentiginous melanomas (ALM). The median tumor thickness was 2.3 mm (0.2–8.5). We observed 18/37 (48.6%) low-risk and 19/37 (51.4%) high-risk primary melanomas. Ulceration was documented in 15/37 (40.5%) primaries. At the time of study beginning, 14/37 (37.8%) were in stage I, 10/37 (27.1%) in stage II, 7/37 (18.9%) in stage III, and 6/37 (16.2%) in stage IV (AJCC 8th edition).

**Fig. 1:**
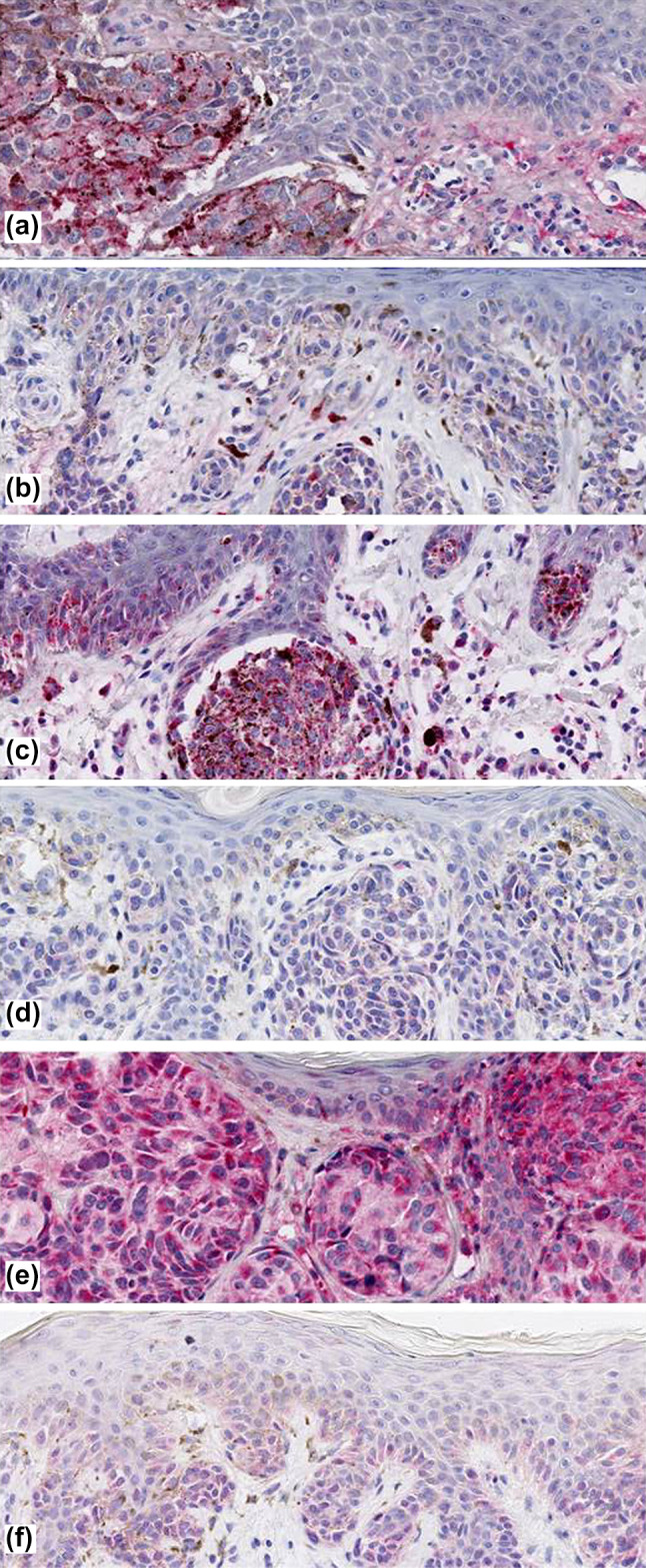
Increased protein expression cutaneous melanoma for COL6A6 **a**, GBP4 **c**, and KLHL41 **e** when compared to benign melanocytic nevi **b**, **d**, **f**

**Fig. 2:**
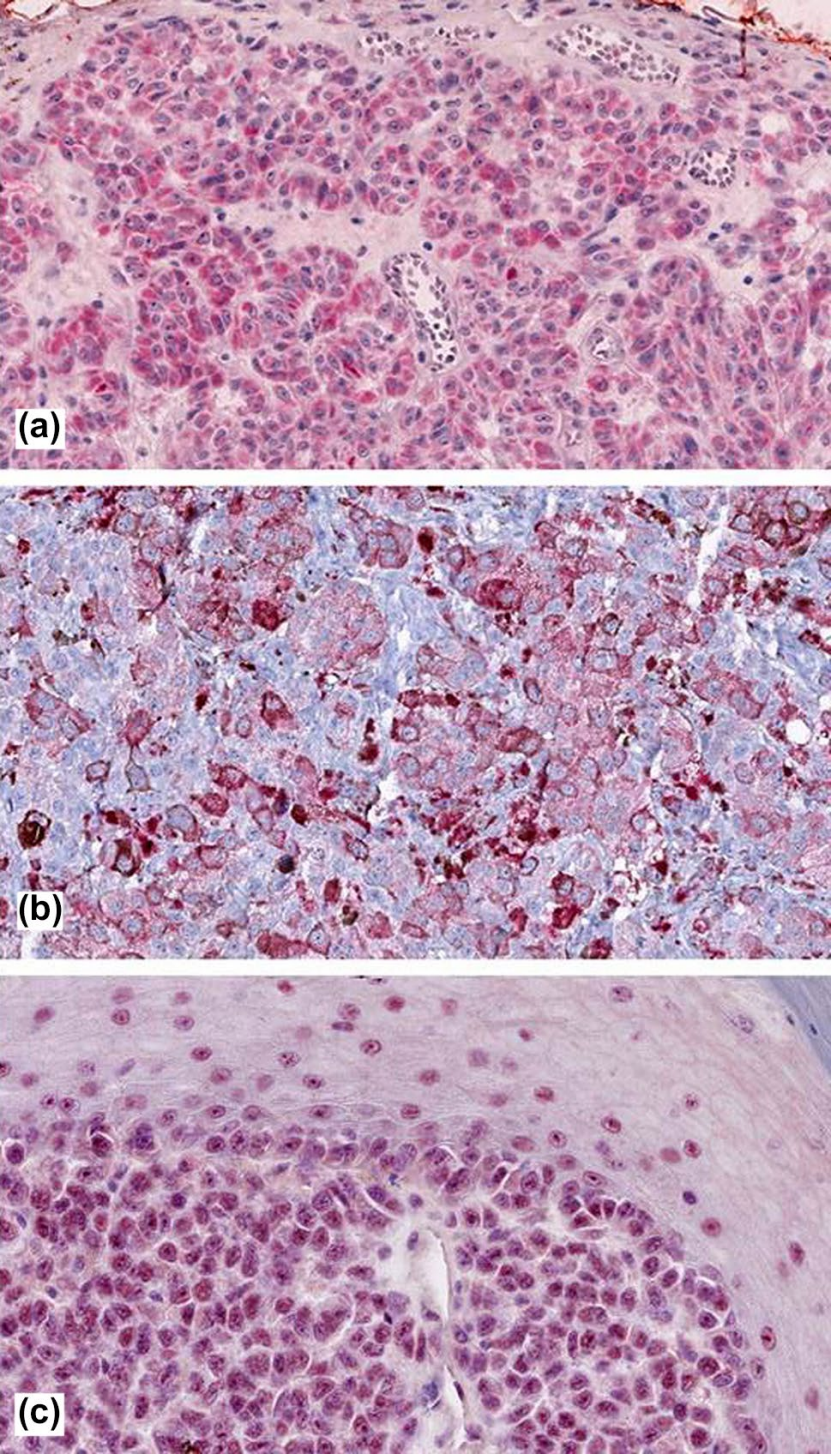
Showing immunoreactivity in cutaneous melanoma for DCD (mainly cytosolic, **a**), KRT9 (membranous, **b**), and PIP (mainly nuclear, **c**)

**Fig. 3:**
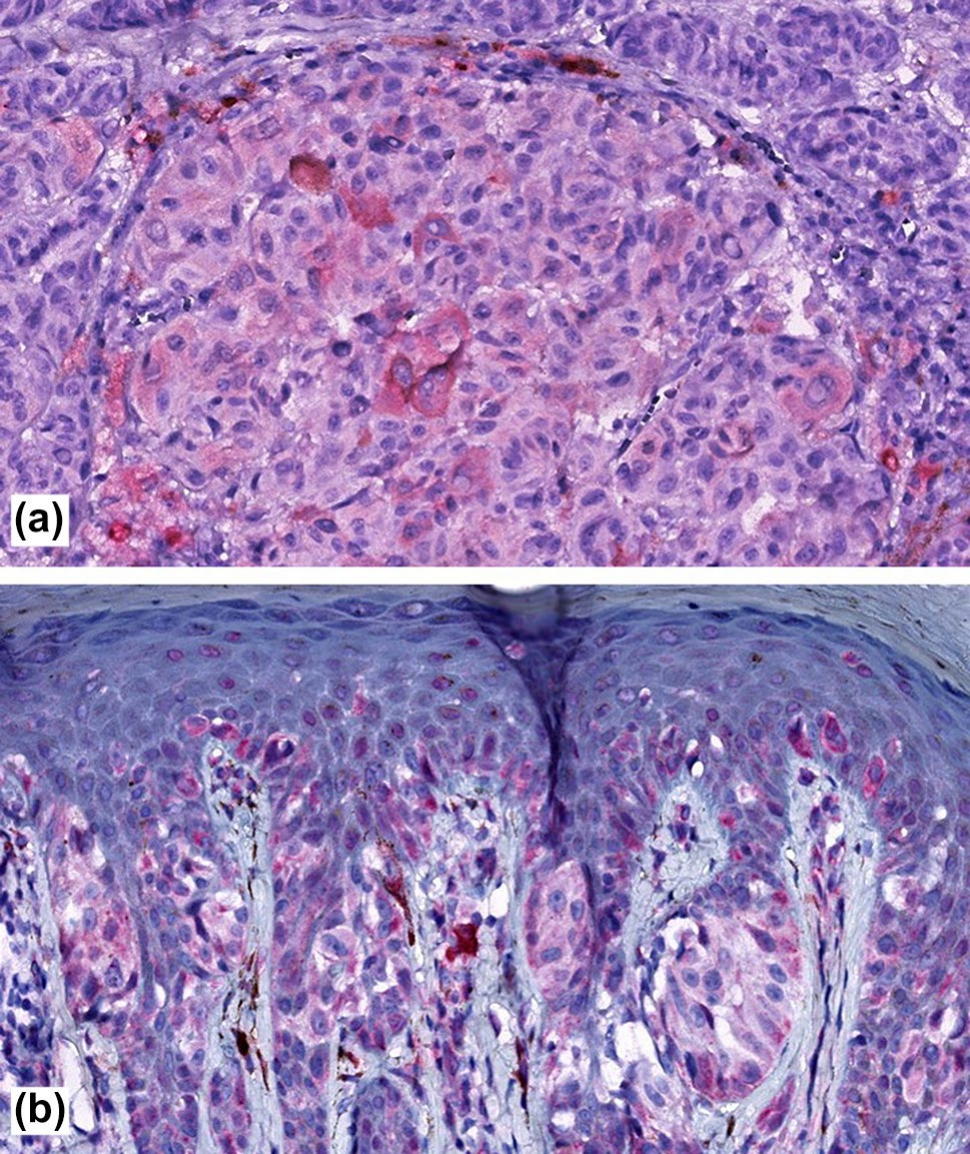
Showing weak predominantly cytosolic immunoreactivity in cutaneous melanoma for SCGB1D2 **a** and SCGB2A2 **b**

Immunohistology staining characteristics of the eight antibodies assessed are detailed in Table 1. Together, the protein expression of most markers investigated was relatively low (e.g., DCD, KRT9, SCGB1D2) and predominantly cytoplasmatic in melanocytes and keratinocytes (Fig. 1, 2, 3). However, immunostaining was also observed in eccrine and sebaceous glands. As demonstrated in Fig. 1 and Table 2, COL6A6 (*P* = 0.0015), GBP4 (*P* = 0.0080), and KLHL41 (*P* = 0.025) expression was significantly enhanced in CM when compared to BN. When evaluating exclusively the melanocytic immunoreactivity, COL6A6 (*P* = 0.0024) expression of CM remained significantly higher as compared to BN. However, GBP4 (*P* = 0.058) and KLHL41 (*P* = 0.061) expression in melanocytes only showed a trend for statistical significance. Expression of the other markers studied did not significantly (*P* > 0.05) differ between CM and BN.

**Table 1:** Immunohistological protein expression characteristics of eight MelaGenix®-relevant genes in cutaneous melanomas and adjacent stroma

Antibody	Stained cell type	Stained cell compartment	Staining localization	Other stained structures/cell types	Other characteristics
COL6α6	Melanocytes	Cytoplasm	Epidermis, dermis	Glands, connective tissue	
DCD	Melanocytes	Cytoplasm	Epidermis, dermis	Glands	Overall very low expression
GBP4	Melanocytes, keratinocytes	Cytoplasm	Epidermis, dermis	Glands, connective tissue	
KLHL41	Melanocytes, keratinocytes	Cytoplasm, partly membranous staining in epidermis	Epidermis, dermis	Glands, connective tissue, blood vessels	More intense staining than the others but partly diffuse
KRT9	Melanocytes, keratinocytes	Membranous, cytoplasm in part	Dermal melanocytes; keratinocytes (horny layer to spinous layer)	Glands	Much more staining in keratinocytes than in melanocytes
PIP	Melanocytes, keratinocytes	In melanocytes nuclear and cytoplasmic staining, keratinocytes rather nuclear staining	Epidermis, dermis	Glands	
SCGB1D2	Melanocytes	Cytoplasm, partly membranous staining	Dermis	Glands	
SCGB2A2	Melanocytes, keratinocytes	Cytoplasm of melanocytes; Nuclear staining in epidermal keratinocytes	Epidermis, dermis	Glands

**Table 2:** Comparison of immunohistochemistry results (H-score) between cutaneous melanoma (CM, *n* = 37) and benign (melanocytic) nevi (BN, *n* = 10): eight proteins have been investigated relating to a prognostic gene expression assay (MelaGenix®) for CM

Antibody	CM H-score *Cases without staining (%) **Only melanocytes	BN H-score *Cases without staining (%) **Only melanocytes	*P* value Mann–Whitney test
COL6A6	23.5 (0–68.2) 3 (8.1) 34.8 (0–110.4)	2.8 (0–25.9) 5 (50) 5 (0–48.8)	= 0.0015 = 0.0024
DCD	0 (0–52.1) 24 (64.9) 0 (0–103.5)	0 (0–13.7) 8 (80) 0 (0–15.6)	n.s.
GBP4	31.5 (9.7–78.3) 0 (0) 48.7 (18.4–128.6)	14.8 (4.8–46.6) 0 (0) 35.5 (9.7–79.8)	= 0.0080 = 0.058***
KLHL41	73.2 (19.1–162) 0 (0) 113.3 (14.8–164.7)	46.1 (18.1–90.9) 0 (100) 74.7 (36.7–155.7)	= 0.025 = 0.061***
KRT9	0 (0–81.4) 22 (59.5) 0 (0–13.9)	0 (0–52.2) 8 (80) 0 (0–70)	n.s.
PIP	60.1 (0–108.3) 1 (2.7) 80.3 (0–166.9)	57.4 (15.1–96.6) 0 (0) 69.8 (8.1–102)	n.s.
SCGB1D2	0 (0–12.8) 28 (75.7) 0 (0–21.5)	0 (0–1.3) 5 (50) 0 (0–1)	n.s.
SCGB2A2	16.7 (0–46.7) 7 (18.9) 25.9 (0–79.6)	19.5 (6.5–47.7) 0 (0) 23.8 (7.3–49.3)	n.s.

*n.s.* non-significant (*P* > 0.1)

*Immunoreactivity > 0

**Only stained melanocytes counted

***Trend for significance

DCD protein expression was significantly correlated with COL6A6 (*r* = 0.37, *P* = 0.023), GBP4 (*r* = 0.49, *P* = 0.0020), and KLHL41 (*r* = 0.35, *P* = 0.037). Moreover, GBP4 was positively correlated with KLHL41 (*r* = 0.40, *P* = 0.014) and was inversely correlated with SCGB2B2 (*r* = − 0.37, *P* = 0.024). Immunoreactivity of proteins studied did not correlate with tumor thickness (P > 0.05) or dichotomized tumor thickness categories (≤ 2 mm vs. > 2 mm, *P* > 0.05). However, SCGB1D2 expression was inversely correlated (*r* = − 0.49, *P* = 0.0023) with serum S100B levels at time of initial CM’s diagnosis. The presence of SCGB1D2 expression was significantly (*P* = 0.043) associated with ulceration of the primary.

KRT9 protein expression was significantly (*P* = 0.0007) more likely found in ALM. Furthermore, the presence of DCD expression was less likely associated with SSM melanoma subtype (*P* = 0.033). The presence of DCD expression was significantly (*P* = 0.020) associated with non-progressive disease (stage I or II) in course of disease. In contrast, the absence of SCGB2A2 expression was significantly (*P* = 0.033) more often observed in patients who had no progress to stage III or IV.

## Discussion

So far, current guidelines do not recommend prognostic gene expression profiling (GEP) assays for CM outside of clinical trials. Nevertheless, their use is becoming more prevalent and some clinicians are already employing GEP assays to manage CM patients. Hence, there is a high need to close the gap between GEP assay use and CM guideline recommendations by gaining high-quality evidence to guide physicians towards the optimal use of GEP testing in CM patients (Grossman et al. 2019). Over the last years, GEP studies of primary CM have been reported in numerous publications (Gambichler et al. 2021a; Amaral et al. 2020; Brunner et al. 2013, 2018; Winnepenninckx et al. 2006; Wardwell-Ozgo et al. 2014; Gschaider et al. 2012; Conway et al. 2009; Rangel et al. 2008; Gerami et al. 2015; Yingjuan et al. 2021; Greenhaw et al. 2020; Garg et al. 2021; Kwak et al. 2020; Eggermont et al. 2020; Wang et al. 2020). Many of the previously reported genes have in common that the cellular source of these mRNAs are not only tumor cells but also endothelial cells (e.g., keratinocytes) and infiltrating lymphocytes etc. (Gambichler et al. 2021a; Amaral et al. 2020; Brunner et al. 2013, 2018; Winnepenninckx et al. 2006; Wardwell-Ozgo et al. 2014; Gschaider et al. 2012; Conway et al. 2009; Rangel et al. 2008; Gerami et al. 2015; Yingjuan et al. 2021; Greenhaw et al. 2020; Garg et al. 2021; Kwak et al. 2020; Eggermont et al. 2020; Wang et al. 2020). Accordingly, in previous expression analyses of the genes used in the MelaGenix® assay, total mRNA was prepared from whole FFPE tissue sections including tumor as well as adjacent tissue. The rationale to include whole tissue sections as opposed to micro- or macro-dissected tumor tissue was the biological significance of the microenvironment and stroma (in particular the tumor/stroma interface) on regulating tumor growth and progression (Weiss et al. 2015). According to this, in the present study we analyzed the protein expression in total and not only in the tumor cells.

Three of the MelaGenix® signature genes (*KRT9*, *KLHL41*, *ECRG2*) have never before been reported to be expressed in melanocytic tumors. For the remaining five genes there exists an overlap with published gene sets allowing the discrimination between advanced and early-stage CM (Winnepenninckx et al. 2006; Ren et al. 2008; Smith et al. 2005). On the basis of hypergeometric distribution calculations, four of the protective genes of the MelaGenix® assay (*DCD*, *PIP*, *SCGB1D2*, *SCGB2A2*) were identified as top 13 of a 50-gene set down-regulated in advanced CM (Brunner et al. 2013; Ren et al. 2008). *DCD*, *PIP*, and *COL6A6* were part of the 254-gene classifiers associated with distant metastasis-free survival of patients with CM (Brunner et al. 2013; Winnepenninckx et al. 2006). Moreover, PIP and SCGB2A2 are listed in several gene sets of a comparative gene expression analysis of CM (Brunner et al. 2013; Greenhaw et al. 2020). Mancuso et al. (Mancuso et al. 2020) showed that in early-stage melanoma patients, serum levels of DCD together with the Breslow thickness are the best predictors of melanoma metastasis. Moreover, Ortega-Martínez et al. (Ortega-Martínez et al. 2016) recently reported that DCD serum levels are elevated in CM patients in regard to healthy subjects, although in early‐stage patients who develop metastasis during follow‐up, DCD levels are significantly decreased. Furthermore, Xu et al. (Xu et al. 2020) analyzed the Pathology Atlas data and found that higher expression of GBP1 and GBP4 was associated with better 5-year survival rate in CM. Accordingly, Yingjuan et al. (Yingjuan et al. 2021) recently proposed a prognostic signature comprising of 10 genes also including GBP4. This 10-gene signature effectively separated CM patients into low- and high-risk groups based upon their survival. Yingjuan et al. (Yingjuan et al. 2021) observed that these low- and high-risk groups also exhibited distinct immune statuses and differing degrees of immune cell infiltration (Yingjuan et al. 2021). Importantly, except for KLHL41, all genes used in MelaGenix® assay are considered protective (Gambichler et al. 2021a; Brunner et al. 2013).

In the present study, we investigated the protein expression of genes included in the commercially available MelaGenix® assay. This study was not designed to focus on the prognostic performance of the protein expression levels assessed, but to describe for the first time the localization, staining characteristics, and expression levels of the proteins relating to the genes of the MelaGenix® assay. Even though the overall expression of the 8 proteins investigated was relatively low, we found statistically significant differences between CM and BN as well as some significant associations with clinical parameters. We have demonstrated that the proteins studied were mainly cytoplasmatically expressed in several cell types, including melanocytes, keratinocytes, and cells of different glands of the skin. For almost all proteins, staining was detected in the epidermis as well as the entire dermis.

We observed that the expression of COL6A6, GBP4, and KLHL41 was significantly higher in CM compared to BN. When evaluating melanocytic protein expression alone, the aforementioned findings could be reproduced in part. The aforementioned findings seem to be in line with the risk-associated role of the corresponding gene in the case of KLHL41. By contrast, COL6A6 and GBP4 have a protective role in CM and a higher protein expression in CM *vs.* BN is unexpected. The expression levels of several proteins (DCD, COL6A6, GBP4) is positively correlated with each other. However, inverse correlations of protein levels were also observed for protective genes which are also positively correlated with the risk-associated gene KLHL41. This finding is difficult to explain given the lack of data on these proteins in CM and their relation to each other. However, we found that SCGB1D2 protein expression is inversely correlated with serum S100B levels at the primary diagnosis, supporting the protective role of *SCGB1D2*. Interestingly, we found that the presence of KRT9 and DCD expression were more likely to be found in non-SSM subtypes. This, in fact, seems like a surprising finding, since the presence of DCD expression was also associated with a more favorable course of disease. As patients with NM and ALM subtypes often present with prognostically unfavorable tumors, one would have expected a different result (Susok and Gambichler 2021; Susok et al. 2021). However, this finding could be partly explained by the proportion of patients with LMM having a relatively good prognosis.

Not all proteins assessed in this study appear to correlate with the protective functions of their corresponding genes. However, the correlation between gene expression and the corresponding protein expression level is a well-known issue, and the presence or absence of such correlation on an individual gene/protein level has been debated in literature for many years (Koussounadis et al. 2015; Edfors et al. 2016). In fact, Koussounadis et al. (2015) recently reported that the profile of correlation coefficients of all genes investigated ranged almost from full −1 (negative correlation) to 1 spectrum (positive correlation). This wide range of coefficients observed in mRNA/protein correlation studies seems perfectly in line with previous studies as well (Koussounadis et al. 2015; Edfors et al. 2016). In conclusion, we have described the protein expression characteristics of 8 corresponding genes recently established for prognostication of the course of disease in CM patients. The protein expression levels observed were relatively low but differed in part with those found in BN. Even though we detected some significant correlations between protein expression levels and clinical parameters (e.g., CM subtype, course of disease), there was no major concordance with the protective or risk-associated functions of the corresponding genes used in the MelaGenix® assay.

## Data Availability

Derived data supporting the findings of this study are available from the corresponding author on reasonable request.

## References

[CR1] Amaral TMS, Hoffmann MC, Sinnberg T, Niessner H, Sülberg H, Eigentler TK, Garbe C (2020) Clinical validation of a prognostic 11-gene expression profiling score in prospectively collected FFPE tissue of patients with AJCC v8 stage II cutaneous melanoma. Eur J Cancer 125:38–45 31838403 10.1016/j.ejca.2019.10.027

[CR2] Brunner G, Reitz M, Heinecke A, Lippold A, Berking C, Suter L, Atzpodien J (2013) A nine-gene signature predicting clinical outcome in cutaneous melanoma. J Cancer Res Clin Oncol 139(2):249–258 23052696 10.1007/s00432-012-1322-z PMC11824186

[CR3] Brunner G, Heinecke A, Falk TM, Ertas B, Blödorn-Schlicht N, Schulze HJ, Suter L, Atzpodien J, Berking C (2018) A prognostic gene signature expressed in primary cutaneous melanoma: synergism with conventional staging. JNCI Cancer Spectr. 2(3):pky032 31360859 10.1093/jncics/pky032 PMC6649804

[CR4] Conway C, Mitra A, Jewell R et al (2009) Gene expression profiling of paraffin-embedded primary melanoma using the DASL assay identifies increased osteopontin expression as predictive of reduced relapse-free survival. Clin Cancer Res 15:6939–6946 19887478 10.1158/1078-0432.CCR-09-1631 PMC2778654

[CR5] Edfors F, Danielsson F, Hallström BM, Käll L, Lundberg E, Pontén F, Forsström B, Uhlén M (2016) Gene-specific correlation of RNA and protein levels in human cells and tissues. Mol Syst Biol 12(10):883 27951527 10.15252/msb.20167144 PMC5081484

[CR6] Eggermont AMM, Robert C (2011) New drugs in melanoma: it’s a whole new world. Eur J Cancer 47:2150–2157 21802280 10.1016/j.ejca.2011.06.052

[CR7] Eggermont AMM, Bellomo D, Arias-Mejias SM, Quattrocchi E, Sominidi-Damodaran S, Bridges AG, Lehman JS, Hieken TJ, Jakub JW, Murphree DH, Pittelkow MR, Sluzevich JC, Cappel MA, Bagaria SP, Perniciaro C, Tjien-Fooh FJ, Rentroia-Pacheco B, Wever R, van Vliet MH, Dwarkasing J, Meves A (2020) Identification of stage I/IIA melanoma patients at high risk for disease relapse using a clinicopathologic and gene expression model. Eur J Cancer 140:11–18 33032086 10.1016/j.ejca.2020.08.029 PMC7655519

[CR8] Gambichler T, Tsagoudis K, Kiecker F, Reinhold U, Stockfleth E, Hamscho R, Egberts F, Hauschild A, Amaral T, Garbe C (2021a) Prognostic significance of an 11-gene RNA assay in archival tissue of cutaneous melanoma stage I-III patients. Eur J Cancer 143:11–18 33278769 10.1016/j.ejca.2020.10.016

[CR9] Gambichler T, Abu Rached N, Tannapfel A, Becker JC, Vogt M, Skrygan M, Wieland U, Silling S, Susok L, Stücker M, Meyer T, Stockfleth E, Junker K, Käfferlein HU, Brüning T, Lang K (2021b) Expression of mismatch repair proteins in Merkel cell carcinoma. Cancers (basel) 13(11):2524 34063983 10.3390/cancers13112524 PMC8196722

[CR10] Garg M, Couturier DL, Nsengimana J, Fonseca NA, Wongchenko M, Yan Y, Lauss M, Jönsson GB, Newton-Bishop J, Parkinson C, Middleton MR, Bishop DT, McDonald S, Stefanos N, Tadross J, Vergara IA, Lo S, Newell F, Wilmott JS, Thompson JF, Long GV, Scolyer RA, Corrie P, Adams DJ, Brazma A, Rabbie R (2021) Tumour gene expression signature in primary melanoma predicts long-term outcomes. Nat Commun 12(1):1137 33602918 10.1038/s41467-021-21207-2 PMC7893180

[CR11] Gerami P, Cook RW, Russell MC et al (2015) Gene expression profiling for molecular staging of cutaneous melanoma in patients undergoing sentinel lymph node biopsy. J Am Acad Dermatol 72:780-785.e783 25748297 10.1016/j.jaad.2015.01.009

[CR12] Gershenwald JE, Scolyer RA, Hess KR et al (2017) Melanoma staging: evidence-based changes in the American Joint Committee on Cancer eighth edition cancer staging manual. CA Cancer J Clin 67(6):472–492 29028110 10.3322/caac.21409 PMC5978683

[CR13] Greenhaw BN, Covington KR, Kurley SJ, Yeniay Y, Cao NA, Plasseraud KM, Cook RW, Hsueh EC, Gastman BR, Wei ML (2020) Molecular risk prediction in cutaneous melanoma: a meta-analysis of the 31-gene expression profile prognostic test in 1479 patients. J Am Acad Dermatol 83(3):745–753 32229276 10.1016/j.jaad.2020.03.053

[CR14] Grossman D, Kim CC, Hartman RI, Berry E, Nelson KC, Okwundu N, Curiel-Lewandrowski C, Leachman SA, Swetter SM (2019) Prognostic gene expression profiling in melanoma: necessary steps to incorporate into clinical practice. Melanoma Manag. 6(4):MMT32. 10.2217/mmt-2019-0016 31871621 10.2217/mmt-2019-0016 PMC6920745

[CR15] Gschaider M, Neumann F, Peters B et al (2012) An attempt at a molecular prediction of metastasis in patients with primary cutaneous melanoma. PLoS ONE 7:e49865 23166783 10.1371/journal.pone.0049865 PMC3498185

[CR16] Koussounadis A, Langdon SP, Um IH, Harrison DJ, Smith VA (2015) Relationship between differentially expressed mRNA and mRNA-protein correlations in a xenograft model system. Sci Rep 5:10775 26053859 10.1038/srep10775 PMC4459080

[CR17] Kwak M, Erdag G, Slingluff CL Jr (2020) Gene expression analysis in formalin fixed paraffin embedded melanomas is associated with density of corresponding immune cells in those tissues. Sci Rep 10(1):18336 33110110 10.1038/s41598-020-74996-9 PMC7591483

[CR18] Long GV, Hauschild A, Santinami M et al (2017) Adjuvant dabrafenib plus trametinib in stage III BRAF-mutated melanoma. N Engl J Med 377(19):1813–1823 28891408 10.1056/NEJMoa1708539

[CR19] Mancuso F, Lage S, Rasero J, Díaz-Ramón JL, Apraiz A, Pérez-Yarza G, Ezkurra PA, Penas C, Sánchez-Diez A, García-Vazquez MD, Gardeazabal J, Izu R, Mujika K, Cortés J, Asumendi A, Boyano MD (2020) Serum markers improve current prediction of metastasis development in early-stage melanoma patients: a machine learning-based study. Mol Oncol 14(8):1705–1718 32485045 10.1002/1878-0261.12732 PMC7400797

[CR20] Ortega-Martínez I, Gardeazabal J, Erramuzpe A, Sanchez-Diez A, Cortés J, García-Vázquez MD, Pérez-Yarza G, Izu R, Luís Díaz-Ramón J, de la Fuente IM, Asumendi A, Boyano MD (2016) Vitronectin and dermcidin serum levels predict the metastatic progression of AJCC I-II early-stage melanoma. Int J Cancer 139(7):1598–1607 27216146 10.1002/ijc.30202 PMC5089559

[CR21] Rangel J, Nosrati M, Torabian S et al (2008) Osteopontin as a molecular prognostic marker for melanoma. Cancer 112:144–150 18023025 10.1002/cncr.23147

[CR22] Ren S, Liu S, Howell P, Xi Y, Enkemann SA, Ju J, Riker AI (2008) The impact of genomics in understanding human melanoma progression and metastasis. Cancer Control 15:202–215 18596672 10.1177/107327480801500303

[CR23] Schadendorf D, Hauschild A (2014) Melanoma—the run of success continues. Nat Rev Clin Oncol 11:75–76 24419300 10.1038/nrclinonc.2013.246

[CR24] Smith AP, Hoek K, Becker D (2005) Whole-genome expression profiling of the melanoma progression pathway reveals marked molecular differences between nevi/melanoma in situ and advanced-stage melanomas. Cancer Biol Ther 4(9):1018–1029 16251803 10.4161/cbt.4.9.2165

[CR25] Susok L, Gambichler T (2021) Caucasians with acral lentiginous melanoma have the same outcome as patients with stage- and limb-matched superficial spreading melanoma. J Cancer Res Clin Oncol. 10.1007/s00432-021-03630-6 33856527 10.1007/s00432-021-03630-6 PMC8800911

[CR26] Susok L, Stücker M, Bechara FG, Stockfleth E, Gambichler T (2021) Multivariate analysis of prognostic factors in patients with nodular melanoma. J Cancer Res Clin Oncol 147(9):2759–2764 33630139 10.1007/s00432-021-03562-1 PMC8310843

[CR27] Wang J, Kong PF, Wang HY, Song D, Wu WQ, Zhou HC, Weng HY, Li M, Kong X, Meng B, Chen ZK, Chen JJ, Li CY, Shao JY (2020) Identification of a gene-related risk signature in melanoma patients using bioinformatic profiling. J Oncol 2020:7526204 32411243 10.1155/2020/7526204 PMC7206882

[CR28] Wardwell-Ozgo J, Dogruluk T, Gifford A et al (2014) HOXA1 drives melanoma tumor growth and metastasis and elicits an invasion gene expression signature that prognosticates clinical outcome. Oncogene 33:1017–1026 23435427 10.1038/onc.2013.30 PMC3982326

[CR29] Weber J, Mandala M, Del Vecchio M et al (2017) Adjuvant nivolumab versus ipilimumab in resected stage III or IV melanoma. N Engl J Med 377(19):1824–1835 28891423 10.1056/NEJMoa1709030

[CR30] Weiss SA, Hanniford D, Hernando E, Osman I (2015) Revisiting determinants of prognosis in cutaneous melanoma. Cancer 121(23):4108–4123 26308244 10.1002/cncr.29634 PMC4666819

[CR31] Winnepenninckx V, Lazar V, Michiels S et al (2006) Gene expression profiling of primary cutaneous melanoma and clinical outcome. J Natl Cancer Inst 98:472–482 16595783 10.1093/jnci/djj103

[CR32] Xu L, Pelosof L, Wang R, McFarland HI, Wu WW, Phue JN, Lee CT, Shen RF, Juhl H, Wu LH, Alterovitz WL, Petricon E, Rosenberg AS (2020) NGS evaluation of colorectal cancer reveals interferon gamma dependent expression of immune checkpoint genes and identification of novel IFNγ induced genes. Front Immunol 11:224. 10.3389/fimmu.2020.00224 32265897 10.3389/fimmu.2020.00224 PMC7103651

[CR33] Yingjuan W, Li Z, Wei C, Xiaoyuan W (2021) Identification of prognostic genes and construction of a novel gene signature in the skin melanoma based on the tumor microenvironment. Medicine (BAltimore) 100(21):e26017. 10.1097/MD.0000000000026017 34032721 10.1097/MD.0000000000026017 PMC8154473

[CR34] Zhu Z, Liu W, Gotlieb V (2016) The rapidly evolving therapies for advanced melanoma—towards immunotherapy, molecular targeted therapy, and beyond. Crit Rev Oncol/hematol. 10.1016/j.critrevonc.2015.12.002 10.1016/j.critrevonc.2015.12.002 26708040

